# Differential patterns of contextual organization of memory in first-episode psychosis

**DOI:** 10.1038/s41537-018-0046-8

**Published:** 2018-02-15

**Authors:** Vishnu P. Murty, Rachel A. McKinney, Sarah DuBrow, Maria Jalbrzikowski, Gretchen L. Haas, Beatriz Luna

**Affiliations:** 1 Department of Psychology, Temple University, Philadelphia, PA USA; 2 Department of Psychology, Carnegie Mellon University, Pittsburgh, PA USA; 3 Princeton Neuroscience Institute, Princeton University, Princeton, NJ USA; 4 Department of Psychiatry, University of Pittsburgh, Pittsburgh, PA USA; 5 VA Pittsburgh Healthcare System, Pittsburgh, PA USA; 6 Department of Psychology, University of Pittsburgh, Pittsburgh, PA USA; 7 Department of Pediatrics, University of Pittsburgh, Pittsburgh, PA USA

## Abstract

Contextual information is used to support and organize episodic memory. Prior research has reliably shown memory deficits in psychosis; however, little research has characterized how this population uses contextual information during memory recall. We employed an approach founded in a computational framework of free recall to quantify how individuals with first episode of psychosis (FEP, *N* = 97) and controls (CON, *N* = 55) use temporal and semantic context to organize memory recall. Free recall was characterized using the Hopkins Verbal Learning Test-Revised (HVLT-R). We compared FEP and CON on three measures of free recall: proportion recalled, temporal clustering, and semantic clustering. Measures of temporal/semantic clustering quantified how individuals use contextual information to organize memory recall. We also assessed to what extent these measures relate to antipsychotic use and differentiated between different types of psychosis. We also explored the relationship between these measures and intelligence. In comparison to CON, FEP had reduced recall and less temporal clustering during free recall (*p* < 0.05, Bonferroni-corrected), and showed a trend towards greater semantic clustering (*p* = 0.10, Bonferroni-corrected). Within FEP, antipsychotic use and diagnoses did not differentiate between free recall accuracy or contextual organization of memory. IQ was related to free recall accuracy, but not the use of contextual information during recall in either group (*p* < 0.05, Bonferroni-corrected). These results show that in addition to deficits in memory recall, FEP differed in how they organize memories compared to CON.

## Psychosis: Memory deficits in first-episode patients

First-episode psychosis patients exhibit impaired memory recall and deviation in how context is used to support recall ability. A US team of researchers led by the University of Pittsburgh’s Vishnu Murty examined how FEP affects an individual’s ability to organize memory based on context, by noting how well patients could recall words from a spoken list. Alongside recollection accuracy, Murty’s team assesed participant ability to recall words said proximally in sequence, and the ability to recall words from the same category—measuring ‘temporal clustering’ and ‘semantic clustering.’ The researchers found that patients with FEP had reduced recall ability and less temporal clustering. Recall accuracy and IQ were also found to be related. This study increases knowledge of FEP-related cognitive changes and could help to target specific therapies.

## Introduction

Impairments in episodic memory, the ability to encode and retrieve details of past experiences, are a core feature of psychosis. Poor episodic memory is among the most reliable deficit in psychosis.^1–4^ These impairments are present across all phases of the illness,^5–7^ and are one of the strongest neurocognitive predictors of conversion to psychosis in high-risk populations.^8^ Characterizing the mechanisms underlying the organization and retrieval of memory in psychosis is critical in order to understand the complex array of cognitive deficits associated with the disorder. In this study, we use an analysis approach inspired by a computational framework of free recall to quantify how individuals in their first episode of psychosis (FEP) differentially use contextual information to support memory recall.

Memories are not stored in isolation; rather, they are organized based on contextual features that are shared across memories.^9^ Individuals tend to recall previous experiences by clustering memories together that share contextual features. Temporal and semantic context are two features that have been shown to prominently organize memory recall. Temporal context reflects features from recent experience that provide a gradually changing internal context,^9^ such that memories encountered close together in time are recalled together (i.e., sequential episodes versus discrete episodes; temporal clustering). Critically, temporal context represents information that reflects recently experienced information and is contextually-relevant. In contrast, semantic context reflects crystalized knowledge about the higher-order organizational structure of how related items are,^10^ and supports the clustering of memories that share pre-existing relationships based on prior knowledge (i.e., cat–dog > cat–hammer; semantic clustering). The context maintenance and retrieval model (CMR) is a computational model that provides quantification of how individuals may use different types of contextual information to guide free recall.^10^ This modeling approach provides insight into distinct processes underlying free recall performance and lets us quantify an individual’s organizational structure of memory to quantify systematic differences across populations. Further, it provides insight into the neural mechanisms underlying free recall, as the use of temporal and semantic context during free recall is associated with the hippocampus and anterior temporal lobe, respectively.^11,12^

Increasing evidence shows that individuals with psychosis show deficits in embedding contextual information in episodic memories. Compared to controls, psychosis patients are more likely to retrieve memories in the absence of contextual details about the event.^1,5,7,13–15^ For example, patients may have equivalent memory for recognizing objects, but show deficits when they have to identify when they saw an object in a sequence of events (i.e., temporal context) or when they have to sort objects into pre-existing categories (i.e., semantic context). In fact, prior research using the CMR framework has shown deficits in using temporal context to support memory recall in chronic schizophrenia.^16^ Open questions remain, however, as to which aspects of memory recall organization underlie memory deficits in individuals with first-episode psychosis. Critically, FEP represent a population who is in close proximity to disease onset without the confounds of prolonged antipsychotic use. Characterizing these contextual deficits in FEP will provide a better mechanistic understanding of patterns of forgetting in this population and could also help target-specific strategies (i.e., semantic versus temporal) to remediate these memory deficits.

In this study, we tested whether FEP (*N* = 97) and controls (CON, *N* = 55) differ in memory accuracy and their use of temporal and semantic context to organize memory recall. Given prior reports that concurrent antipsychotic use may influence the neural circuitry underlying episodic memory,^17,18^ we further tested whether antipsychotic use affects the accuracy or contextual organization of memory recall. In addition, we tested whether deficits in the contextual organization of memory relative to normative controls were specific to schizophrenia-related disorders or were transdiagnostic across psychosis. Finally, given a prior report that IQ may account for differences in memory organization in chronic schizophrenia,^16^ we evaluated the relationship of IQ with memory performance.

## Results

### Accuracy and contextual organization of free recall in FEP vs. CON

**Fig. 1:**
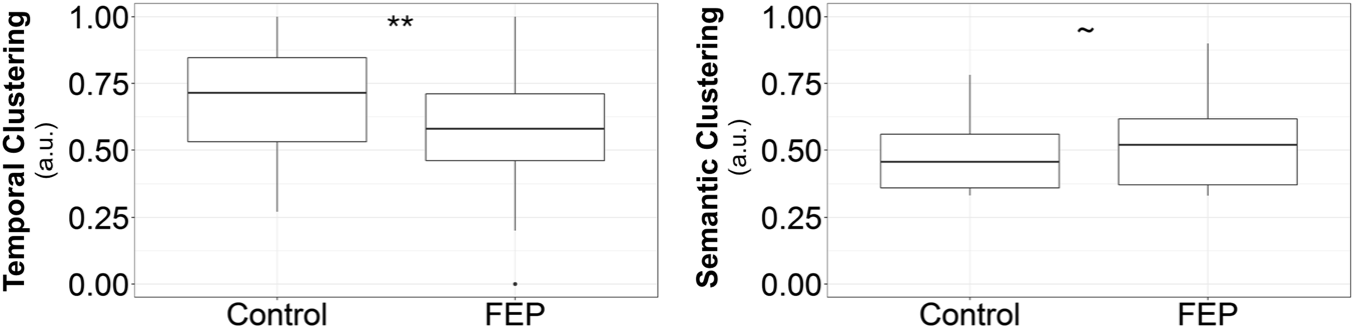
Contextual influences on Memory Recall Organization. CON use temporal context to a greater extent during free recall than FEP (Left). FEP use semantic context to a greater extent during free recall then CON (Right). In the box-and-whisker plots the horizontal line represents the median, the edges of the box indicate the upper and lower quartiles, and the median is represented by the solid horizontal lines, the edges of box indicate the upper and lower quartiles, and the vertical lines represent the range (excluding outliers which are indicated by a dot). Asterisks (**) indicates significance of *p* < 0.001, Bonferroni-corrected; ~ indicates a trend of *p* ≤ 0.10, Bonferroni-corrected

The FEP group exhibited reduced free recall accuracy in comparison to CON (% recalled (standard error, se); CON: 62.6 (2.0); FEP: 52.3 (1.6); difference in accuracy: *ß* ± se = −0.07 ± .027, *p* = 0.03, corrected, Table 1). FEP also utilized temporal clustering less often than CON (*p* = 0.02, corrected; Fig. 1, Left; Table 1). FEP also tended to show greater semantic clustering compared to CON, however, these results were only trend level when correcting for multiple comparisons (*p* = 0.03, uncorrected; *p* = 0.10; corrected; Fig. 1, Right; Table 1). This prior analysis only investigated semantic clustering on the first trial of learning, and the use of semantic clustering may change with multiple study episodes (i.e., multiple trials of learning). When analyzing all three trials of learning, there was a main effect of group such that FEP used semantic clustering more often then CON (*p* = 0.05, Supplementary Table 1), however, there was no interaction between group and trial number (*p* = 0.37, Supplementary Table 1). Notably, all of the above analyses showed there was no influence of memory performance (Table 1, Supplementary Table 1), and the same pattern of results remained when age and sex were not included as covariates (Supplementary Table 2).

**Table 1:** Results from general linear models comparing free recall accuracy and organization across FEP and CON

Summary of free recall accuracy and organization			
	Items recalled	Temporal	Semantic
	Mean (SD)	Mean (SD)	Mean(SD)
FEP	6.28 (1.99)	0.59 (0.20)	0.52 (0.14)
CON	7.51 (1.80)	0.70 (0.19)	0.48 (0.12)
	beta	se	*p*
# of Items recalled			
Constant	0.3223	0.0897	0.001
FEP vs. CON	−0.0706	0.0274	0.03
Age	0.0018	0.0029	1
Sex	−0.0198	0.0247	1
PSES	0.0043	0.0009	<0.001
Education (yrs)	0.0067	0.0061	0.82
Temporal clustering			
Constant	0.7181	0.1203	0
FEP vs. CON	−0.1006	0.036	0.02
Age	−0.0066	0.0037	0.23
Sex	−0.0039	0.0318	1
PSES	−0.0004	0.0013	1
Education (yrs)	0.0197	0.0079	0.04
Recall accuracy	−0.2003	0.1065	0.19
Semantic clustering			
Constant	0.4418	0.0826	0
FEP vs. CON	0.053	0.0247	0.1
Age	−0.0011	0.0025	1
Sex	0.001	0.0219	1
PSES	0.0004	0.0009	1
Education (yrs)	−0.0079	0.0054	0.43
Recall accuracy	0.2546	0.0731	0.002

*p*-values reflect Bonferonni-corrected scores

*SD* standard deviation, *SE* standard error, *yrs* years

### Accuracy and contextual organization of free recall within FEP

Antipsychotic-naive patients (*N* = 45) did not differ from those on antipsychotics (*N* = 52) in free recall accuracy, temporal clustering, or semantic clustering (all *p*’s > 0.40, Supplementary Table 2). Similarly, schizophrenia spectrum patients (*N* = 59) did not perform different from patients with other psychotic disorders (*N* = 38, Supplementary Table 3) on all free recall measures (all *p*’s > 0.30, uncorrected). Notably, this pattern of significance did not differ when we removed age and sex from the model (Supplementary Table 3–4).

There were no relationships between any of our measures of free recall performance with negative symptoms, positive symptoms, or global functioning (all *p*’s > 0.17, uncorrected).

### Relationships between accuracy and contextual organization in FEP vs. CON

To test whether there were relationships between free recall accuracy and use of contextual organization, we ran simple regressions between accuracy and strategy use in each group separately (FEP, CON). We did not find any significant relationships between temporal clustering and free recall accuracy in either group (FEP: *r*(96) = −0.15, *p* = 0.46, uncorrected; CON: *r*(54) = −0.10, *p* = 0.52, uncorrected). While there was no relationship between semantic clustering and accuracy in CON (*r*(54) = 0.24, *p* = 0.07, uncorrected), there was a significant relationship between semantic clustering and recall accuracy in FEP (*r*(96) = −0.30, *p* < 0.05, corrected).

### Relationships with general intelligence and free recall performance

To test whether patterns of free recall performance across FEP and CON persisted when correcting for intelligence, we included IQ into our statistical models. When controlling for IQ, CON continued to show greater free recall accuracy (*p* = 0.03, corrected), greater use of temporal clustering (*p* = 0.04, corrected), and less use of semantic clustering (*p* = 0.05, corrected) compared to FEP (Supplementary Table 5). This pattern of significance did not differ when we removed age and sex from the model (Supplementary Table 5).

**Fig. 2:**
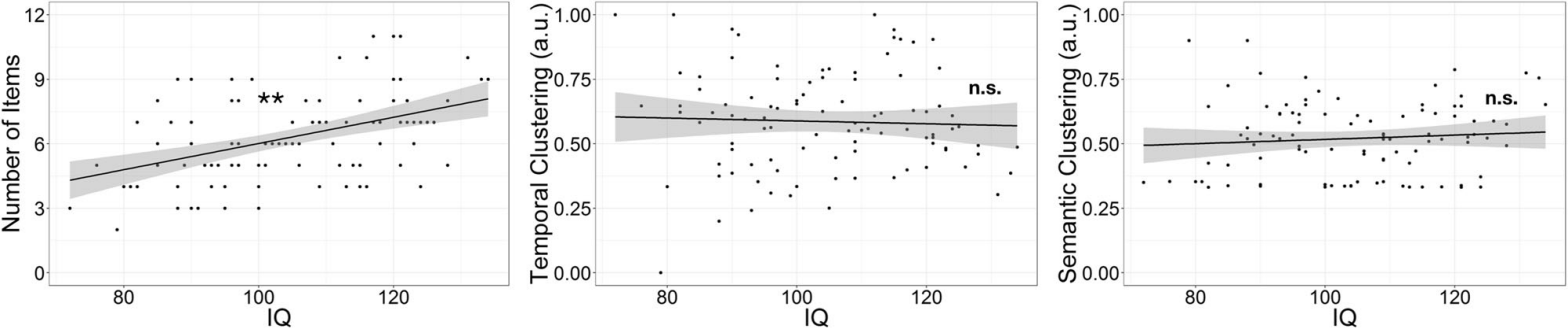
IQ relates to free recall accuracy but not use of temporal or semantic context in FEP. There was a significant relationship between IQ (left) and free recall accuracy in FEP. However, there was no relationship with IQ (middle/right) and the use of temporal or semantic context during free recall. The solid line indicates the best linear fit and the shaded area represents the 95% confidence interval. Asterisks (**) indicates significance of *p* < 0.001, Bonferonni-corrected; n.s indicates *p* > 0.10, uncorrected

To directly test relationships between IQ and free recall performance, we ran simple regressions in our FEP and CON groups, separately (Fig. 2). We found significant relationships of IQ with free recall accuracy in both CON (*r*(54) = 0.45, *p* < 0.001, corrected) and FEP (*r*(96) = 0.45, *p* < 0.001, corrected; Fig. 2). Unlike recall accuracy, there was no relationship between IQ and temporal clustering or semantic clustering in CON (all *p*’s > 0.7, uncorrected) or FEP (all *p*’s > 0.4, uncorrected; Fig. 2). Our findings suggest that while free recall accuracy may reflect some influence of general intellectual function, measures of temporal and semantic clustering are measures of memory performance that are relatively unbiased with respect to IQ.

## Discussion

In the current study, we examined how individuals with FEP use contextual information to organize memory recall. By employing analyses inspired by a computational framework of retrieved context,^10^ we characterized differences in free recall accuracy as well as how temporal and semantic context influence the spontaneous organization of free recall. We found that FEP recalled fewer words and were less likely to use temporal context to organize free recall compared to CON. Further, we found a trend towards FEP using semantic context to a greater extent than CON to organize free recall. Patterns of memory recall performance did not differ as a function of concurrent antipsychotic use, and were similar in schizophrenia-spectrum versus other psychotic disorders. Finally, we found that IQ was related to free recall accuracy but, not in how context is used to organize free recall.

While accounting for general deficits in recall accuracy, we found that FEP were less likely to use temporal context to organize memories compared to CON. Normative individuals may use contextual details acquired during learning to organize memories, whereas, FEP may recall previous experience with diminished access to contextual information about the learning episode (i.e., nearby items in a word list). This suggests that during recall, when memories are retrieved in psychosis, there is less information about what events also happened in close temporal proximity. Our findings dovetail well with prior research showing deficits in temporal order memory in psychosis, in which patients had worse performance in determining when a single event happened in a sequence of events.^19,20^ Together, these findings suggest that individuals with psychosis have deficits in binding temporal information into memory, resulting in the retrieval of information isolated from the context in which it was presented. Given that many adaptive behaviors, such as statistical inference, credit assignment, and forward planning, rely on knowledge of the temporal order of events,^21–24^ deficits in temporal context may have broad impacts on executive function in individuals with psychosis.

In addition to temporal context deficits, we found a trend towards FEP using semantic information more often to organize memory recall. FEP relied upon semantic categories more often during recall compared to CON both on the first trial and across all three trials. Interestingly, prior research has documented that individuals with schizophrenia show deficits in semantic memory and semantic fluency, while still maintaining intact knowledge on semantic structures.^25–28,29^ A recent study also suggested that patients have intact implicit activation of semantic structures, but fail to activate semantic context in a task-appropriate manner.^30^ In our study, FEP were not encouraged to organize information semantically, but may have relied on semantic information given deficits in utilizing temporal context. We hypothesize that controls may use semantic information more often than FEP when it directly benefits performance. In the current study, semantic clustering only benefitted FEP and not CON. Future research is necessary, however, to determine how different learning contexts may differentially engage the use of semantic context in FEP.

Finally, we found that while memory recall accuracy was related to IQ in both groups, IQ was not related to the use of contextual information to organize memory recall. Interestingly, a previous study demonstrated that chronic schizophrenia patients were less likely to use temporal context to organize free recall, but those context deficits were fully accounted for by differences in IQ.^16^ Our divergent results show that in FEP, which is closer to the proximity in disease onset, differences in using context to organize free recall are independent of general intelligence. However, extended use of medication and chronic illness may lead to generalized deficits that affect both temporal context and generalized intelligence. Thus, our results provide important evidence for a core deficit in recall accuracy independent of increasing impairments through the clinical course of psychosis.

There were a few limitations of the current cohort that limit the interpretation of our findings. First, the IQ of the FEP population in the current cohort did not differ from the CON. In most samples, FEP individuals have lower baseline IQ compared to matched controls.^31^ In many ways equivalent IQ across groups may be a benefit as it removes a potential confound, however, it will be critical to determine if the same pattern of memory performance we report generalize to FEP populations with lower baseline IQ. Second, our current sample consisted of individuals who were either medication naïve or on less than 2-months lifetime treatment of antipsychotics. While this permitted a comparison of how concurrent antipsychotic use influences memory performance, our sample is ill-suited to investigate how antipsychotic treatment influences memory. Future work examining individuals on longer doses of antipsychotic treatment or using longitudinal designs would better characterize the role of antipsychotics on memory recall accuracy and organization in FEP.

In this study, we characterized the use of contextual information to organize free recall in FEP. We provide evidence that impaired temporal processing in psychosis may play a primary role in known recall impairments in the disorder. These findings extend prior literatures documenting memory in psychosis by highlighting differences not only in the content of memory (i.e., accuracy), but also in the organizational structure of memory. Importantly, this pattern of results suggests that in psychosis, memories are may be organized based on internal knowledge frameworks (i.e., semantics), rather then veridical experiences (i.e., temporal context). This reliance on internal versus external knowledge may represent a core feature of thought disturbances, as internal knowledge frameworks may be aberrant in the disorder. Notably, our results indicating a specific impairment in temporal processing support models that posit hippocampal impairment in psychosis, though this still needs to be directly tested. Finally, understanding how individuals experiencing a first episode of psychosis organize their memories may point to pathway to remediate memory deficits in this population. Specifically, interventions can build upon prior remediation protocols that utilize repetitions and sequences^32^ to focus on enhancing patients’ ability to bind temporal information to memoranda, as well as capitalize upon utilizing prior semantic knowledge to facilitate memory.

## Methods

### Participants

Participants were recruited from the inpatient and outpatient services of the Western Psychiatric Institute and Clinic (WPIC) and extensively evaluated using medical, neurological, and psychiatric assessments. From a total sample of 181 participants, 29 participants were removed for missing neuropsychological testing/clinical assessment (*N* = 21), incomplete data collection (*N* = 5), missing demographic information (*N* = 3). The final sample included 97 FEP patients and 55 matched controls (CON). Demographic information for FEP and CON groups is presented in Table 2. All study participants were provided with written informed consent according to the guidelines of the University of Pittsburgh Institutional Review Board and all procedures were performed in accordance the University of Pittsburgh Institutional Review Board and the Declaration of Helsinki. All participants or their legal guardians provided written informed consent after study procedures were fully explained and were compensated for participation.

**Table 2:** Demographic and clinical information for the final sample

	First-episode psychosis (*N* = 97)	Controls (*N* = 55)	*p*-value
Mean age (SD) in years	22.85 (5.4)	22.89 (5.0)	0.96
Age range	12–40	14–41	
Sex	57 M/40 F	30 M/25 F	0.62
Race	46 W/42 B/9 O	35 W/16 B/4 O	0.15
Mean IQ (SD)	104.3 (14.7)	105.6 (11.1)	0.56
Mean parental socioeconomic status (SD)	40.51 (13.4)	45.24 (13.6)	0.04
Mean education (SD) in years	12.46 (2.4)	14.31 (2.6)	<0.001
# of FEP on antipsychotics	52	–	–
Mean chlorapromazine equivalent FEP on antipsychotics (SD)	269.7 mg (357.1)	–	–
Positive symptoms (BPRS [range])	13.57 (5–24)	–	–
Negative symptoms (BPRS [range])	6.58 (3–12)	–	–

Concurrent anti-psychotic dosage was converted into chlorapromazine equivalents.^39^

*SD* standard deviation, *W* white, *B* black, *O* other, *mg* milligrams

Exclusion criteria for all participants included: significant neurological disorder, head injury, or medical illness affecting the central nervous system function, IQ (determined using the Wechsler Abbreviated Scale of Intelligence, ref. ^33^) lower than 75, DSM-IV substance dependence or substance abuse disorder within the prior 6 months; or any contraindications for use of MRI, given that some of the participants were also involved in neuroimaging studies. Inclusion criteria for FEP were as follows: first episode of psychotic symptoms and antipsychotic-naive or prescribed antipsychotic treatment for less than two months. The patients on medication (*N* = 52) included individuals on risperidone (*N* = 27), olanzapine (*N* = 7), aripiprazole (*N* = 5), haloperidol (*N* = 3), queitiapine (*N* = 2), haloperidol + risperidone (*N* = 3), olanzapine + risperidone (*N* = 2), aripiprazole + queitiapine (*N* = 1), haloperidole + risperidone + olanzapine (*N* = 2), and aripiprazole + haloperidol + risperidone (*N* = 1). Diagnoses were determined using all available clinical information and data gathered from a Structured Clinical Interview for DSM-IV (SCID, ref. ^34^) conducted by a trained Masters-level or PhD-level clinician. Senior diagnosticians/clinical researchers (including GLH) arrived at a consensus diagnosis at diagnostic conferences in which all available clinical data were reviewed. The patient sample was separated into two groups: schizophrenia spectrum (schizophrenia, schizophreniform, or schizoaffective disorder diagnosis) and other psychotic disorders (affective psychosis or psychotic disorder not otherwise specified). Illness duration for each patient was also determined in the consensus conference after a review of historical information about psychosis onset.

The inclusion criteria for controls were no lifetime history of a major psychiatric disorder or antipsychotic treatment, no first-degree family member with a history of a psychotic disorder.

### Cognitive assessments

Free recall data for the current study were extracted from the Hopkins verbal learning test—revised.^35^ The HVLT-R is an orally administered test with three identical trials in which a trained experimenter reads out loud to the participant a list of 12 words from three semantic categories (animals, dwellings, and precious stones) at a pace of 1 word per second. Each participant received the same list of words, presented in the same order across all trials. Immediately following each presentation of the list, the participant was asked to recall as many words as possible in any order. The experimenter recorded the order of responses. Following Polyn and colleagues,^16^ for all of our main analyses investigating free recall accuracy, temporal context and semantic context we only analyzed data from the first trial of three, so as to not confound our measures with multiple learning contexts as well as retrieval contexts. However, we include an additional post-hoc analysis investigating semantic clustering over all three trials.

In addition, the Wechsler Abbreviated Scale of Intelligence (WASI, Wechsler 1999) was administered to all participants to obtain a standardized, full-scale intelligence quotient (IQ). FSIQ was estimated using two subtests, vocabulary and matrix reasoning.

### Clinical measures

Severity of clinical symptoms was rated using the Brief Psychiatric Rating Scale (BPRS; see ref. ^36^^,37^). The BPRS includes 18 self-report and observational items pertaining to somatic concerns, anxiety, emotional withdrawal, conceptual disorganization, guilt feelings, tension, mannerisms and posturing, grandiosity, depressive mood, hostility, suspiciousness, hallucinatory behaviors, motor retardation, uncooperativeness, unusual thought content, blunted affect, excitement, and disorientation over the past seven-days. Trained clinicians administered the BPRS,^36^ rating individual items on a seven-point scale (1, not present; 2, very mild; 3, mild; 4, moderate; 5, moderately sever; 6, sever; 7, extremely sever). For the purposes of this study, we used the total negative and positive symptom scale scores (BPRS-NEG and BPRS-POS^38^).

### Data analysis

We quantified three measures of free recall performance from the HVLT-R: recall accuracy, temporal clustering, and semantic clustering. Recall accuracy was defined as the number of words successfully recalled from the word list divided by the total number of words in the list (i.e., 12). We then derived individual subject measures of how temporal and semantic context contributed to the organization of free recall based on CMR.^10^ To quantify contributions of temporal context, we calculated a temporal clustering score. Temporal clustering measured an individual’s tendency to transition in recall to words that were contiguous with the just-recalled-word (i.e, items that were presented in close temporal proximity to a recalled item). To quantify temporal clustering, for each recalled word, the remaining possible words are ranked according to their temporal distance from the just-recalled word. Then, a percentile score is given to the subsequently recalled word given these probabilities. Then, these percentile scores are averaged across all transitions. This procedure normalizes the score depending on how many possible recalls are available. Values closer to 1 indicated that participants were recalling words in sequential order (forward or backwards), whereas values closer to .5 indicated that participants were randomly ordering the sequence of words during free recall. To quantify contributions of semantic context, we calculated a semantic clustering score. To quantify semantic clustering, for each recalled word, the remaining possible words are scored according to whether they were from the same or different category from the just-recalled world (a 1 for the same category, a 0 for a different). Subsequently, the percentile scores are averaged across all transitions. Values closer to 1 indicate recalling based on semantic category, whereas values closer to .5 would indicate that semantic information was not organizing free recall. Data were analyzed using the Behavioral Toolbox Release 1.1 (http://memory.psych.upenn.edu/Behavioral_toolbox).

To determine if there were group differences, we constructed general linear models (GLMs) using GLMFIT as implemented in MATLAB R2016a. We ran separate GLMs on the free recall, temporal clustering, and semantic clustering data. In each model, the independent variable of interest was group (FEP vs. CON) while controlling for age, sex, years of education, and PSES. For the analysis of temporal and semantic clustering, we also controlled for individual’s free recall accuracy (total number of words recalled). Finally, given that the use of semantic clustering may change over repeated trials, we ran an additional analysis investigating group differences (FEP vs. CON) over the three trials, while controlling for age, sex, years of education, and PSES. Notably, for all of the above models the same pattern of results was obtained when we did not include age or sex in our statistical models.

Within the FEP group, we conducted another set of general linear models to determine if there was a significant effect of antipsychotic medication use (antipsychotic-naive vs. medicated) on our free recall measures. Critically, the intent of this analysis was to determine if concurrent use of anti-psychotic medications influenced free recall performance. We additionally investigated within the FEP group whether there was a a diagnostically specific effect of psychotic disorder diagnosis (schizophrenia-spectrum vs. other psychotic disorders).

To determine if our measures of free recall related to clinical and functional outcomes in FEP, we computed bivariate correlations between free recall measures (proportion recalled, temporal clustering, and semantic clustering) with BPRS-POS, BPRS-NEG, and GAF scores.

To determine if there were relationships between free recall accuracy and clustering strategy, we computed bivariate correlations of free recall accuracy with temporal and semantic clustering, respectively. Finally, to determine the putative role of general intellectual function in episodic memory function, we computed bivariate correlations between free recall performance and IQ in each group.

For all analyses, results were considered significant at a Bonferroni-corrected *p*-value < 0.05. For our main comparisons across groups (FEP vs. CON), we corrected for three statistical comparisons (accuracy, temporal clustering, and semantic clustering) yielding an adjusted *p*-value of *p* < 0.0167. For the post-hoc comparisons of semantic clustering and trial number, there was only one comparison. For each of our within group comparisons (antipsychotic-naïve vs. medicated; schizophrenia-spectrum vs. other psychotic disorder), we corrected for three statistical comparisons (accuracy, temporal clustering, and semantic clustering) yielding an adjusted *p*-values of *p* < 0.0167. We corrected for four statistical comparisons for our correlation analyses between free recall accuracy and clustering, because there were two measures (temporal clustering, semantic clustering) investigated separately in each group (CON, FEP) yielding an adjusted *p*-value of *p* < 0.0125. We corrected for six statistical comparisons for our correlation analyses between IQ and free recall measures, because there were three measures (proportion recalled, temporal contiguity, semantic contiguity) investigated separately in each group (CON, FEP) yielding an adjusted *p*-value of *p* < 0.0083.

### Data availability

The data that support the findings of this study are available from the corresponding author upon reasonable request.

## Electronic supplementary material

Supplementary Table 1

Supplementary Table 2

Supplementary Table 3

Supplementary Table 4

Supplementary Table 5
