# Supplementary material for: Differential patterns of contextual organization of memory in first-episode psychosis
Source: NPJ Schizophr. 2018 Feb 15;4:3. doi: 10.1038/s41537-018-0046-8 (PMC5814439; doi:10.1038/s41537-018-0046-8)
Supplement: Supplementary file 1 — Supplementary Table 1 [file 41537_2018_46_MOESM1_ESM.docx]

Supplementary Table 1: Comparisons of FEP and CON on semantic clustering across trials

| **w/ age and sex as covariates** | |  |  |
| --- | --- | --- | --- |
|  | beta | se | p |
| constant | 0.38224 | 0.052495 | <0.001 |
| FEP vs CON | 0.063755 | 0.033114 | 0.05 |
| trial | -0.0011184 | 0.013235 | 0.93 |
| age | 0.0029837 | 0.0014225 | 0.04 |
| sex | -0.0038794 | 0.012245 | 0.75 |
| PSES | 0.0015599 | 0.00049311 | 0.002 |
| education (yrs) | -0.011501 | 0.0032161 | <0.001 |
| recall accuracy | 0.016267 | 0.0035539 | <0.001 |
| group*trial | -0.013843 | 0.015577 | 0.37 |
|  |  |  |  |
| **w/o age and sex as covariates** | |  |  |
|  | beta | se | p |
| constant | 0.40053 | 0.04914 | <0.001 |
| FEP vs CON | 0.070129 | 0.03315 | 0.04 |
| trial | -0.00043435 | 0.0133 | 0.97 |
| PSES | 0.0013264 | 0.00048264 | 0.006 |
| education (yrs) | -0.0076679 | 0.0026501 | 0.004 |
| recall accuracy | 0.016148 | 0.0035703 | <0.001 |
| group*trial | -0.014037 | 0.01566 | 0.37 |
|  |  |  |  |
