# Supplementary material for: Differential patterns of contextual organization of memory in first-episode psychosis
Source: NPJ Schizophr. 2018 Feb 15;4:3. doi: 10.1038/s41537-018-0046-8 (PMC5814439; doi:10.1038/s41537-018-0046-8)
Supplement: Supplementary file 2 — Supplementary Table 2 [file 41537_2018_46_MOESM2_ESM.docx]

Supplementary Table 2: Comparisons of FEP and CON without age and sex as covariates

| **# of Items Recalled** | |  |  |
| --- | --- | --- | --- |
|  | beta | se | p |
| constant | 0.311 | 0.0792 | 0.0004 |
| FEP vs CON | -0.0666 | 0.0268 | 0.04 |
| PSES | 0.0042 | 0.0009 | <0.001 |
| education (yrs) | 0.0087 | 0.005 | 0.24 |
|  |  |  |  |
| **Temporal Clustering** | |  |  |
|  | beta | se | P |
| constant | 0.6527 | 0.1079 | <0.001 |
| FEP vs CON | -0.1128 | 0.0354 | 0.005 |
| PSES | 0.0002 | 0.0013 | 1 |
| education (yrs) | 0.0117 | 0.0065 | 0.23 |
| recall accuracy | -0.2078 | 0.1066 | 0.16 |
|  |  |  |  |
| **Semantic Clustering** | |  |  |
|  | beta | se | p |
| constant | 0.4338 | 0.0733 | <0.001 |
| FEP vs CON | 0.0509 | 0.0241 | 0.11 |
| PSES | 0.0005 | 0.0009 | 1 |
| education (yrs) | -0.0092 | 0.0044 | 0.12 |
| recall accuracy | 0.2531 | 0.0724 | 0.002 |
