# Supplementary material for: Differential patterns of contextual organization of memory in first-episode psychosis
Source: NPJ Schizophr. 2018 Feb 15;4:3. doi: 10.1038/s41537-018-0046-8 (PMC5814439; doi:10.1038/s41537-018-0046-8)
Supplement: Supplementary file 3 — Supplementary Table 3 [file 41537_2018_46_MOESM3_ESM.docx]

Supplementary Table 3: Comparisons of FEP on and off anti-psychotics

| **Temporal Clustering** (w/ age + sex as covariates) | | |  |
| --- | --- | --- | --- |
|  | beta | se | P |
| constant | 0.5387 | 0.1325 | <0.001 |
| antipsychotic use | -0.0147 | 0.041 | 1 |
| age | -0.0004 | 0.0053 | 1 |
| sex | 0.0073 | 0.0418 | 1 |
| PSES | 0.0012 | 0.0018 | 1 |
| education (yrs) | 0.0111 | 0.0128 | 1 |
| recall accuracy | -0.256 | 0.1332 | 0.17 |
|  |  |  |  |
| **Semantic Clustering** (w/ age + sex as covariates) | | |  |
|  | beta | se | p |
| constant | 0.548 | 0.0892 | <0.001 |
| antipsychotic use | 0.0255 | 0.0276 | 1 |
| age | -0.0034 | 0.0036 | 1 |
| sex | -0.0027 | 0.0281 | 1 |
| PSES | 0.0008 | 0.0012 | 1 |
| education (yrs) | -0.0112 | 0.0086 | 0.58 |
| recall accuracy | 0.2821 | 0.0897 | 0.006 |
|  |  |  |  |
| **# of Items Recalled** (w/o age + sex as covariates) | | |  |
|  | beta | se | p |
| constant | 0.2592 | 0.0868 | 0.01 |
| antipsychotic use | -0.0307 | 0.032 | 1 |
| PSES | 0.0039 | 0.0013 | 0.008 |
| education (yrs) | 0.0099 | 0.0072 | 0.51 |
|  |  |  |  |
| **Temporal Clustering** (w/o age + sex as covariates) | | | |
|  | beta | se | P |
| constant | 0.5468 | 0.1144 | 0 |
| antipsychotic use | -0.0145 | 0.0404 | 1 |
| PSES | 0.0012 | 0.0017 | 1 |
| education (yrs) | 0.0107 | 0.0091 | 0.73 |
| recall accuracy | -0.2592 | 0.1305 | 0.15 |
|  |  |  |  |
| **Semantic Clustering** (w/o age + sex as covariates) | | | |
|  | beta | se | p |
| constant | 0.5204 | 0.0774 | 0 |
| antipsychotic use | 0.0273 | 0.0273 | 0.96 |
| PSES | 0.0012 | 0.0011 | 0.81 |
| education (yrs) | -0.017 | 0.0062 | 0.02 |
| recall accuracy | 0.2819 | 0.0883 | 0.006 |
