# Supplementary material for: Differential patterns of contextual organization of memory in first-episode psychosis
Source: NPJ Schizophr. 2018 Feb 15;4:3. doi: 10.1038/s41537-018-0046-8 (PMC5814439; doi:10.1038/s41537-018-0046-8)
Supplement: Supplementary file 4 — Supplementary Table 4 [file 41537_2018_46_MOESM4_ESM.docx]

Supplementary Table 4: Comparisons of schizophrenia-spectrum and other psychosis

| **# of Items Recalled** (w/ age + sex as covariates) | | |  |
| --- | --- | --- | --- |
|  | beta | se | p |
| constant | 0.3161 | 0.1021 | 0.008 |
| psychosis group | -0.0223 | 0.0327 | 1 |
| age | 0.0015 | 0.0042 | 1 |
| sex | -0.0444 | 0.0327 | 0.53 |
| PSES | 0.004 | 0.0014 | 0 |
| education (yrs) | 0.007 | 0.0099 | 1 |
|  |  |  |  |
| **Temporal Clustering** (w/ age + sex as covariates) | | |  |
|  | beta | se | P |
| constant | 0.5434 | 0.1361 | <0.001 |
| psychosis group | -0.0108 | 0.0416 | 1 |
| age | -0.0004 | 0.0053 | 1 |
| sex | 0.0066 | 0.0418 | 1 |
| PSES | 0.0012 | 0.0018 | 1 |
| education (yrs) | 0.0107 | 0.0126 | 1 |
| recall accuracy | -0.2539 | 0.133 | 0.18 |
|  |  |  |  |
| **Semantic Clustering** (w/ age + sex as covariates) | | |  |
|  | beta | se | p |
| constant | 0.5634 | 0.092 | <0.001 |
| psychosis group | -0.0113 | 0.0281 | 1 |
| age | -0.0038 | 0.0036 | 0.87 |
| sex | -0.0028 | 0.0283 | 1 |
| PSES | 0.0008 | 0.0012 | 1 |
| education (yrs) | -0.0096 | 0.0085 | 0.7911 |
| recall accuracy | 0.2717 | 0.0898 | 0.001 |
|  |  |  |  |
| **# of Items Recalled** (w/o age + sex as covariates) | | |  |
|  | beta | se | p |
| constant | 0.2671 | 0.0888 | 0.01 |
| psychosis group | -0.0214 | 0.0324 | 1 |
| PSES | 0.0039 | 0.0013 | 0.007 |
| education (yrs) | 0.0088 | 0.0071 | 0.65 |
|  |  |  |  |
| **Temporal Clustering** (w/o age + sex as covariates) | | | |
|  | beta | se | P |
| constant | 0.5503 | 0.1168 | <0.001 |
| psychosis group | -0.0108 | 0.0407 | 1 |
| PSES | 0.0013 | 0.0017 | 1 |
| education (yrs) | 0.0101 | 0.009 | 0.79 |
| recall accuracy | -0.257 | 0.1302 | 0.15 |
|  |  |  |  |
| **Semantic Clustering** (w/o age + sex as covariates) | | | |
|  | beta | se | p |
| constant | 0.5306 | 0.0794 | <0.001 |
| psychosis group | -0.007 | 0.0277 | 1 |
| PSES | 0.0013 | 0.0011 | 0.77 |
| education (yrs) | -0.016 | 0.0061 | 0.03 |
| recall accuracy | 0.2717 | 0.0885 | 0.009 |
