# Supplementary material for: Differential patterns of contextual organization of memory in first-episode psychosis
Source: NPJ Schizophr. 2018 Feb 15;4:3. doi: 10.1038/s41537-018-0046-8 (PMC5814439; doi:10.1038/s41537-018-0046-8)
Supplement: Supplementary file 5 — Supplementary Table 5 [file 41537_2018_46_MOESM5_ESM.docx]

Supplementary Table 5: Comparisons of FEP and CON when including IQ as a covariate

| **# of Items Recalled** (w/ age + sex as covariates) | | | |
| --- | --- | --- | --- |
|  | beta | se | p |
| constant | 0.0257 | 0.1078 | 1 |
| FEP vs CON | -0.0815 | 0.0259 | 0.006 |
| age | 0.0009 | 0.0027 | 1 |
| sex | -0.0126 | 0.0233 | 1 |
| pses | 0.0027 | 0.001 | 0.02 |
| education (yrs) | 0.0016 | 0.0058 | 1 |
| iq | 0.0043 | 0.001 | <0.001 |
|  |  |  |  |
| **Temporal Clustering** (w/ age + sex as covariates) | | | |
|  | beta | se | p |
| constant | 0.727 | 0.1478 | <0.001 |
| FEP vs CON | -0.0999 | 0.0367 | 0.02 |
| age | -0.0066 | 0.0037 | 0.24 |
| sex | -0.0041 | 0.032 | 1 |
| pses | -0.0003 | 0.0014 | 1 |
| education (yrs) | 0.0198 | 0.008 | 0.04 |
| recall accuracy | -0.1962 | 0.1138 | 0.26 |
| iq | -0.0001 | 0.0014 | 1 |
|  |  |  |  |
| **Semantic Clustering** (w/ age + sex as covariates) | | | |
|  | beta | se | p |
| constant | 0.4645 | 0.1014 | <0.001 |
| FEP vs CON | 0.0547 | 0.0252 | 0.09 |
| age | -0.001 | 0.0026 | 1 |
| sex | 0.0006 | 0.0219 | 1 |
| pses | 0.0005 | 0.0009 | 1 |
| education (yrs) | -0.0076 | 0.0055 | 0.51 |
| recall accuracy | 0.2651 | 0.0781 | 0.003 |
| iq | -0.0004 | 0.001 | 1 |
|  |  |  |  |
| **# of Items Recalled** (w/o age + sex as covariates) | | | |
|  | beta | se | p |
| constant | 0.0123 | 0.0994 | 1 |
| FEP vs CON | -0.0796 | 0.0253 | 0.006 |
| pses | 0.0026 | 0.0009 | 0.01 |
| education (yrs) | 0.0025 | 0.0049 | 1 |
| iq | 0.0043 | 0.001 | <0.001 |
|  |  |  |  |
| **Temporal Clustering** (w/o age + sex as covariates) | | | |
|  | beta | se | p |
| constant | 0.6697 | 0.1376 | <0.001 |
| FEP vs CON | -0.1114 | 0.0362 | 0.008 |
| pses | 0.0003 | 0.0013 | 1 |
| education (yrs) | 0.012 | 0.0067 | 0.23 |
| recall accuracy | -0.1998 | 0.1142 | 0.25 |
| iq | -0.0003 | 0.0014 | 1 |
|  |  |  |  |
| **Semantic Clustering** (w/o age + sex as covariates) | | | |
|  | beta | se | p |
| constant | 0.4577 | 0.0934 | <0.001 |
| FEP vs CON | 0.0529 | 0.0246 | 0.09 |
| pses | 0.0005 | 0.0009 | 1 |
| education (yrs) | 0.2643 | 0.0775 | 0.003 |
| recall accuracy | -0.0087 | 0.0046 | 0.17 |
| iq | -0.0004 | 0.001 | 1 |
